# Case Report: Extrinsic Compression of the Left Main Coronary Artery by a Giant Sinus of Valsalva Aneurysm

**DOI:** 10.3389/fcvm.2021.753154

**Published:** 2021-11-15

**Authors:** Feifei Ning, Manyun Tang, Mengjie Wang, Joseph B. Muhlestein, John D. Day, Guoliang Li, Yang Yan

**Affiliations:** ^1^Department of Cardiovascular Medicine, The First Affiliated Hospital of Xi'an Jiaotong University, Xi'an, China; ^2^Department of Cardiology, Intermountain Heart Institute, Murray, UT, United States; ^3^Department of Cardiology, St. Mark's Hospital, Salt Lake City, UT, United States; ^4^Department of Cardiovascular Surgery, The First Affiliated Hospital of Xi'an Jiaotong University, Xi'an, China

**Keywords:** angina pectoris, syncope episodes, sinus of Valsalva aneurysm, surgical treatment, myocardial ischemia, case report

## Abstract

Sinus of Valsalva aneurysm (SoVA) is an uncommon clinical entity, which is present in roughly 0. 09% of the general population. The cause can either be acquired or congenital. Clinically the SoVA of unruptured status are rarely captured or even diagnosed due to atypical clinical presentations. Here, we present a rare case of exertional angina pectoris and recurrent syncope due to an extrinsically compressed left coronary artery by a giant SoVA in a 50-year-old female patient. This SoVA was successfully repaired by the surgical exclusion and the patient was still doing well after 2 years of follow-up.

## Introduction

Sinus of Valsalva aneurysm (SoVA) is an enlargement of the aortic root area between the aortic valve annulus and the sinotubular ridge (1) that is caused by weakness of the elastic lamina at the junction of the aortic media and the annulus fibrosis. SoVAs can be congenital or acquired. Trauma, tuberculosis, syphilis, and bacterial endocarditis can contribute to their formation. Unfortunately, most SoVAs are missed or misdiagnosed due to atypical clinical presentations. In this study, we present a patient who complained of syncope episodes with a history of exertional angina pectoris. The physical examination and laboratory data of the patient were unremarkable. Imaging studies done indicated that it could be an extrinsically compressed left coronary artery by a giant SoVA causing her symptoms. Following successful surgery, there was complete resolution of all symptoms.

## Case Description

**Figure 1:**
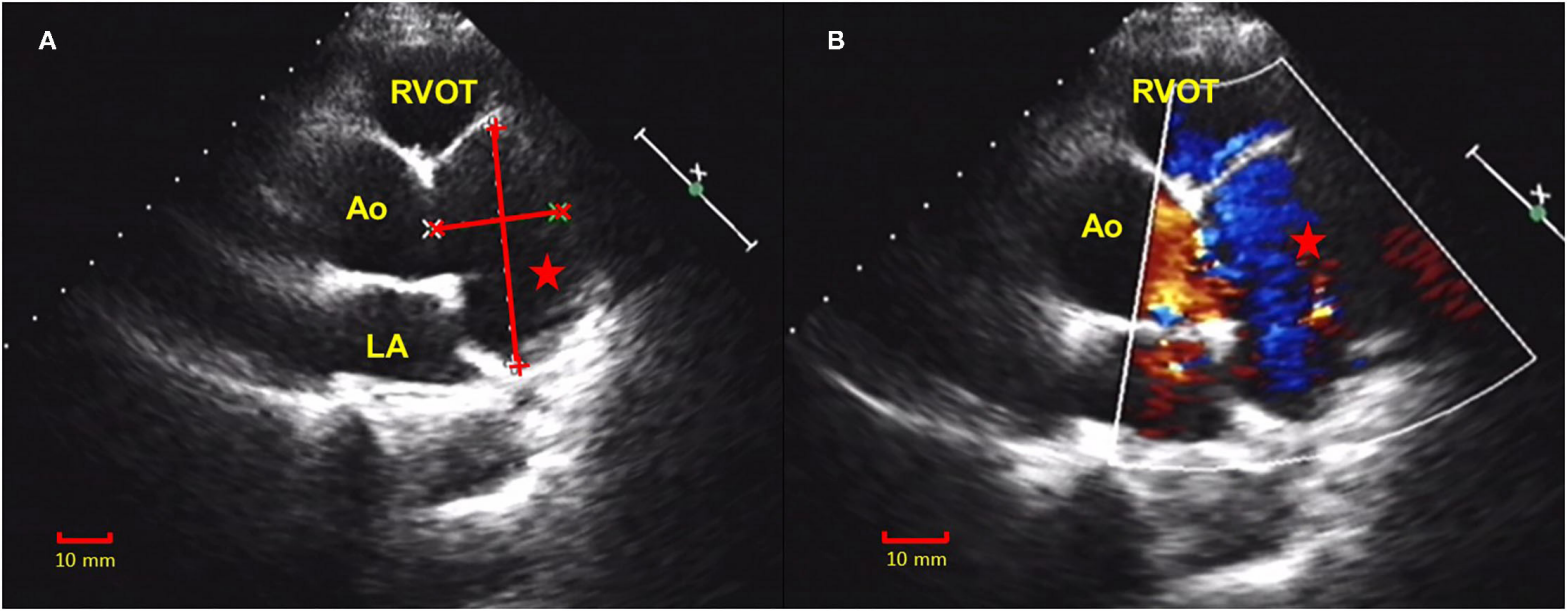
Transthoracic echocardiogram (TTE) showed a giant aneurysm at the parasternal aortic short axis view that was originated from the left sinus of Valsalva **(A)**. Communication of the blood flow was detected between the aneurysm and the aorta **(B)**. TTE, transthoracic echocardiogram; RVOT, right ventricle outflow tract; Ao, aorta; LA, left atrium; red star, sinus of Valsalva aneurysm (SoVA).

**Figure 2:**
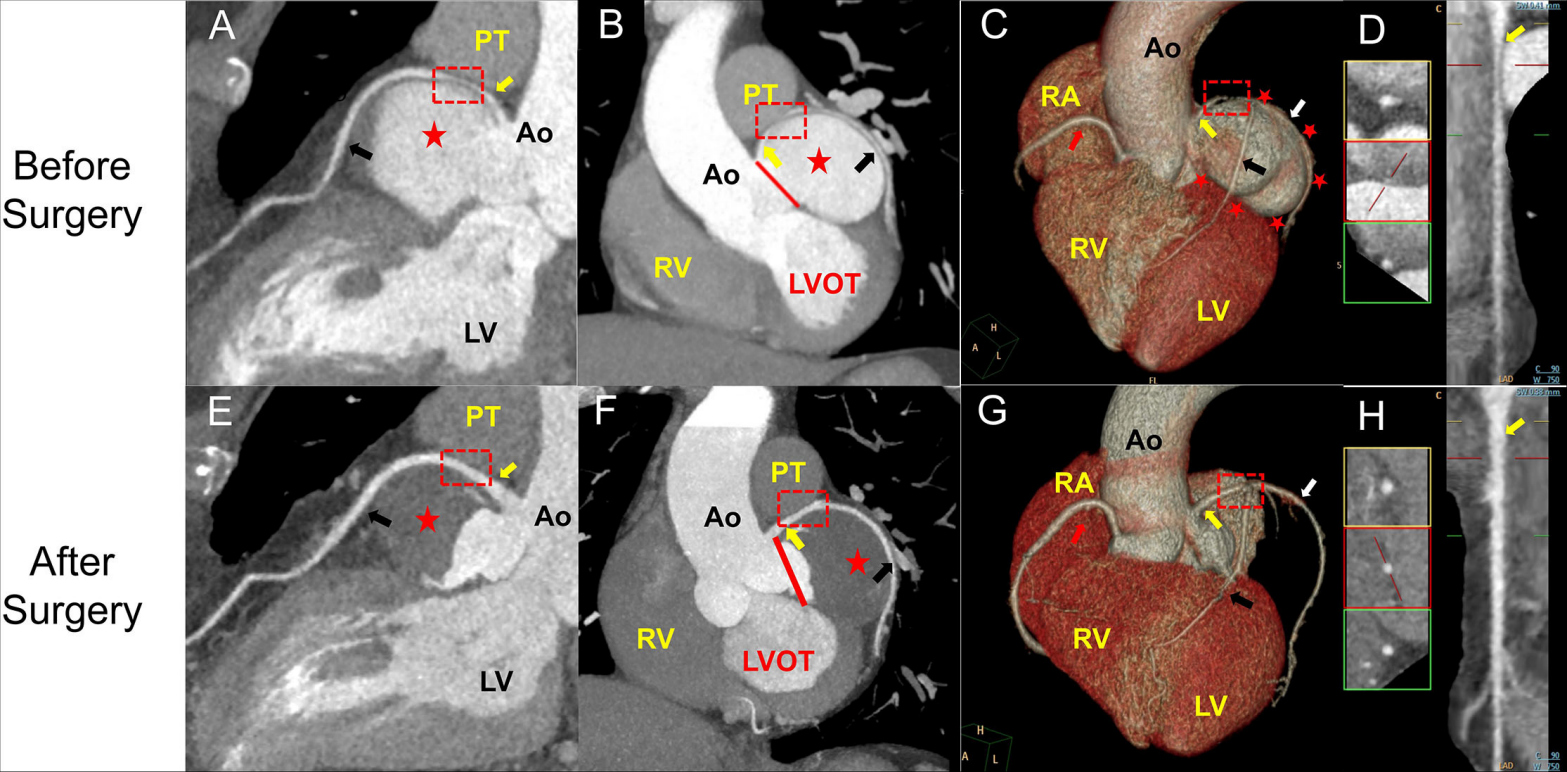
Coronary computed tomography angiogram (CCTA) images of the SoVA obtained before **(A–D)** and after **(E–H)** surgical treatment. **(A,E)** show a continuous curved vessel reconstruction of the left main and left anterior descending coronary arteries based on enhanced spiral CT before **(A)** and after the surgery **(E)**. **(B,E)** show the CT coronal section of the reconstructed heart before **(B)** and after **(F)** the surgical exclusion. **(C)** shows a three-dimensional reconstruction model of the aneurysm before surgery, and **(G)** shows the same view absent the aneurysm after the surgical exclusion. **(D,H)** show the long-axis curvilinear reformation of the left main coronary artery (LMCA) before **(D)** and after the surgery **(H)**. LMCA is identified with a red rectangle demonstrating extrinsic compression before surgery that has resolved after. The PT and LA were eliminated in **(C,F)** to show the LMCA. LMCA, left main coronary artery (yellow arrow); LAD, left anterior descending coronary artery (black arrow); PT, pulmonary trunk; LV, left ventricle; Ao, aorta; RV, right ventricle; LVOT, left ventricle outflow tract; LV, left ventricle; red star, SoVA; red line, the neck of the aneurysm; red arrow, right coronary artery; white arrow, left circumflex coronary artery.

**Figure 3:**
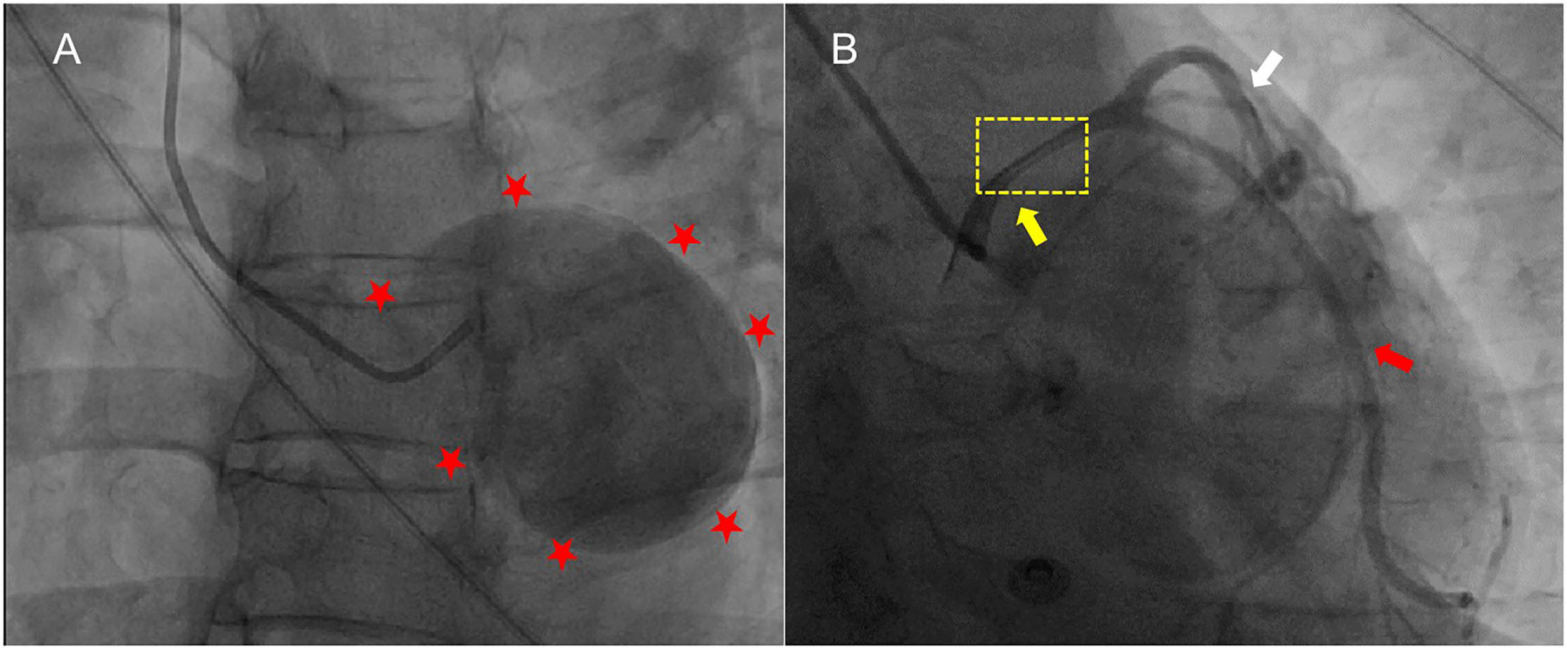
The CAG showed the severely dilated left sinus of Valsalva and the contrast agent was preserved and rotated in a circle **(A)**. The aneurysm compressed the LMCA (yellow arrow) in the upward direction with significant LMCA seriously narrowing **(B)**. No critical stenosis was observed in LAD. Red stars, the outline of the SoVA; CAG, coronary artery angiography; LMCA, left main coronary artery; LAD, left anterior descending coronary artery, black arrow; white arrow, left circumflex coronary artery.

A 50-year-old female was presented with a history of exertional angina pectoris for 8 months and recurrent syncope for 2 weeks. Her physical examination and laboratory data were unremarkable. Electrocardiogram (ECG) showed no sign suggestive of myocardial ischemia. The transesophageal echocardiography (TEE) showed a giant aneurysm at the parasternal aortic short axis view, which originated from the left sinus of Valsalva (Figure 1A). Communication of blood flow was detected between the aneurysm and the aorta (Figure 1B). The coronary computed tomography angiogram (CCTA) showed a giant left SoVA (54.4 mm × 46.9 mm × 60.4 mm) extending posteriorly and inferiorly, and located on the upper left of the left ventricular outflow tract. The left main coronary artery (LMCA) opening originated from the neck of the aneurysm, and its proximal portion extended above the body of the aneurysm (Figures 2A–D). Coronary angiography (CAG) confirmed these findings by showing persistent swirling of contrast within the dilated aneurysm (Figure 3; Supplementary Video 1).

**Figure 4:**
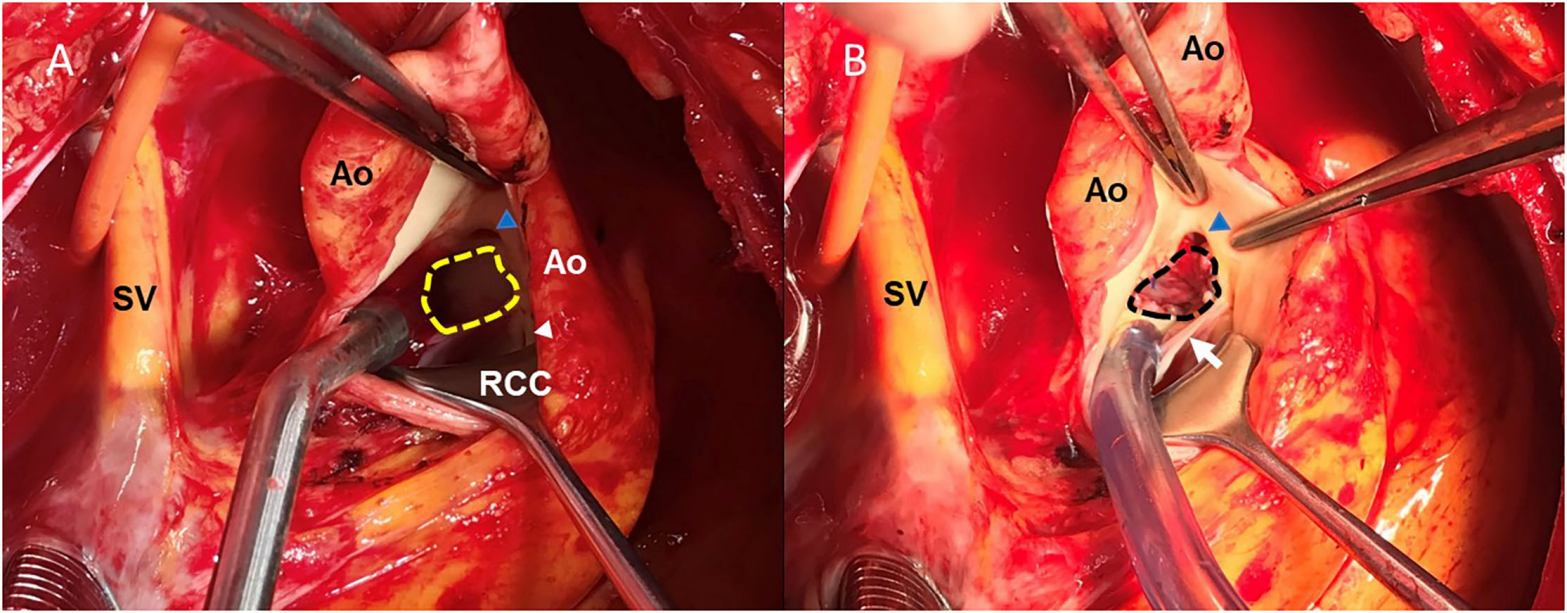
The gross view of the aneurysm *in situ* initially observed before **(A)** and after **(B)** the surgical exclusion. The neck of the aneurysm originated from the left sinus of Valsalva (shown in the yellow-dotted border), and the LMCA originated from the LCC, just above the neck of the aneurysm **(A)**. A patch (shown in black-dotted border) was used to close the opening of the aneurysm, thereby, reconstructing the left coronary sinus **(B)**. LMCA, left main coronary artery; Ao, aorta; SV, superior vena cava; LCC, left coronary cusp; RCC, right coronary cusp; blue triangle, the opening of the LCMA; white triangle, the opening of the right coronary artery; white arrow, left and right coronary valve.

Anatomically, the aneurysm squeezed the LMCA in the upward direction, causing significant extrinsic compression between the body of the aneurysm and the pulmonary trunk and presumably acting as the principal source of the symptoms of the patient with angina pectoris and syncope. Accordingly, the patient underwent surgical exclusion of the aneurysm and placement of a heart patch (Figure 4). Postoperative CCTA showed that the SoVA was not enhanced during the computed tomography angiogram and the blood flow through the LMCA recovered significantly, with the percentage diameter stenosis that decreased from 62% to 21% (Figures 2E–H). The percentage diameter stenosis was calculated by dividing the diameter of the compressed LMCA by the average of the proximal and distal reference vessel diameter. The patient had an uneventful postoperative recovery and was still doing well after 2 years of follow-up.

## Discussion

The sinus of the Valsalva aneurysm is an extremely rare entity that is associated with the enlargement of the aortic root area between the aortic valve annulus and the sinotubular ridge. Sinus of Valsalva aneurysms are found in roughly 0.09% of the general population and are usually diagnosed as an incidental finding or after an acute rupture into an adjacent cardiac structure (2). Generally, men are more likely to be affected, and there is a higher reported incidence in Asian groups (3). SoVAs are mainly associated with the right coronary sinus, followed by the non-coronary sinus, and, rarely, by the left coronary sinus (4). Patients with the SoVAs may have different clinical presentations ranging from complete asymptomatic to cardiogenic shock or sudden cardiac death. Sinus of Valsalva aneurysms are often missed until they rupture into the cardiac chambers, causing acute clinical symptoms (5).

Clinical manifestations of Sinus of Valsalva aneurysms are atypical, SoVAs could be present with dyspnea, palpitations, or angina, they can occasionally cause heart block, atrial fibrillation, obstruction of cardiac outflow, and even compress the coronary arteries, thereby causing myocardial infarction or angina (6). Ischemic heart disease, including angina pectoris and ST-elevation myocardial infarction are rare manifestations of SoVAs (7). Syncope might occur as a result of severe arrhythmias or hypotension occurring as a result of myocardial ischemia. An extremely rare cause of myocardial ischemia is the SoVA that can impair coronary circulation by compressing the epicardial coronary artery and disturbing the blood flow (7).

As unruptured SoVAs are in general asymptomatic and difficult to diagnose, these are often found inadvertently during an echocardiogram examination and cardiac catheterization. Many imaging modalities can be utilized in the diagnosis of a SoVA. Transthoracic and transesophageal echocardiogram are the standard imaging technique for this lesion. CCTA has been increasingly utilized and it is also useful to imaging patients who are at low-to-intermediate risk for coronary artery disease prior to surgical repair.

Ruptured SoVAs require surgical intervention, however, in the setting of a non-ruptured SoVA, treatment should be based on the specific situation of the patient. Surgical repair should also be considered in an enlarging SoVA or an unruptured but symptomatic SoVA, such as outflow tract obstruction, arrhythmia, or infection.

## Conclusion

In this study, we report a case of a giant unruptured aneurysm arising from the left sinus of the Valsalva Sinus, which led to severe extrinsic compression of the left main coronary artery and resulted in angina and syncope secondary to the coronary ischemia. All symptoms resolved after the surgical exclusion. The SoVA is an infrequent clinical entity that has only rarely been reported to be associated with syncope and angina. This case represents an uncommon cause of syncope and angina raises the discussion of the appropriate imaging and treatment strategies of of SoVAs.

## Data Availability Statement

The original contributions presented in the study are included in the article/Supplementary Material, further inquiries can be directed to the corresponding authors.

## Ethics Statement

Written informed consent was obtained from the individual(s) for the publication of any potentially identifiable images or data included in this article.

## Author Contributions

FN, MT, and YY drafted the manuscript and contributed to the case collection. MW, JM, and JD provided figures and formalized the manuscript. YY and GL reviewed the drafts and approved the final manuscript as submitted. All the authors approved the submitted version.

## Funding

This work was supported by Institutional Foundation of the First Affiliated Hospital of Xi'an Jiaotong University, China (2018QN-05).

## Conflict of Interest

The authors declare that the research was conducted in the absence of any commercial or financial relationships that could be construed as a potential conflict of interest.

## Publisher's Note

All claims expressed in this article are solely those of the authors and do not necessarily represent those of their affiliated organizations, or those of the publisher, the editors and the reviewers. Any product that may be evaluated in this article, or claim that may be made by its manufacturer, is not guaranteed or endorsed by the publisher.
